# Computational Modeling and Analysis of Microarray Data: New Horizons

**DOI:** 10.3390/microarrays5040026

**Published:** 2016-10-21

**Authors:** Heather J. Ruskin

**Affiliations:** Sci-Sym Centre (Scientific Computing & Complex Systems Modelling), School of Computing, Dublin City University, Dublin 9, Ireland; Heather.Ruskin@dcu.ie; Tel.: +353-1-700-5513

**Keywords:** microarray data types, high-throughput genomic analysis, modeling gene expression, augmentation, integration, sequencing techniques

## Abstract

High-throughput microarray technologies have long been a source of data for a wide range of biomedical investigations. Over the decades, variants have been developed and sophistication of measurements has improved, with generated data providing both valuable insight and considerable analytical challenge. The cost-effectiveness of microarrays, as well as their fundamental applicability, made them a first choice for much early genomic research and efforts to improve accessibility, quality and interpretation have continued unabated. In recent years, however, the emergence of new generations of sequencing methods and, importantly, reduction of costs, has seen a preferred shift in much genomic research to the use of sequence data, both less ‘noisy’ and, arguably, with species information more directly targeted and easily interpreted. Nevertheless, new microarray data are still being generated and, together with their considerable legacy, can offer a complementary perspective on biological systems and disease pathogenesis. The challenge now is to exploit novel methods for enhancing and combining these data with those generated by alternative high-throughput techniques, such as sequencing, to provide added value. Augmentation and integration of microarray data and the new horizons this opens up, provide the theme for the papers in this Special Issue.

## Editorial

Microarray technologies have been in the forefront of managing large amounts of genomic data over an extended period and have evolved with need. The history of microarray development was traced by Bumgarner [1] in a brief review which highlights major landmarks, from early colony hybridization methods and the first recognizable DNA microarrays of ordered arrays in the late 1970s through to reusable filters, cDNA synthesis, the ability to clone arrays and new methods of production and fluorescence detection. More recently, advances in sequencing methods have led to determination of full gene sets, focusing on targeted DNA sub-sequences and complete genome typing for some organisms. The various production technologies have sought to address issues of reproducibility, expense, customization, quality and others for a wide range of applications; the most common being measurement of gene expression levels. As the first true high-throughput technologies for genomics, the generation of large microarray datasets also led to issues of quality and the potential for sharing and motivated development of the MIAME (Minimum Information About a Microarray Experiment) standard in the early 2000s. Nevertheless, despite the availability of public databases, limitations such as technology differences, concentration range reliability, multiple or omitted genes, with concomitant ‘noise’ issues for data analysis and interpretation, have persisted. Reduced costs, associated with second and even third generation sequencing methods, with direct relation of sequence counts to species concentration and less noisy data, currently pose a direct challenge to the continued use of microarrays. Given the enormous legacy inherent in microarray data, however, it is important to gauge what these still have to offer to genomics’ research in the intermediate and longer term. 

Unarguably, biological and medical fields are now data-rich, to a degree that was unknown prior to high-throughput techniques and genome-wide methods, but reconciliation of different generation technologies, inconsistency in data gathering and type, data volume, as well as heterogeneous and often incompatible storage formats, pose non-trivial problems. Nonetheless, incorporating biological information from diverse data can yield important benefits in terms of improved insight to organism function and disease development. To this end, much effort in recent years has focused on building tools and adapting statistical analyses to enhance value and facilitate integration of different data types. 

The papers in this Special Issue reflect a number of aspects of this effort, from novel augmentation of microarray data to derivation of a framework and methods for combined analyses of data from different sources. Enhancement at the initial stage of data generation is the focus of the article by Jaimes-Díaz et al. [2]. The authors evaluate genomic fingerprints for *Bacillus anthracis*, obtained by virtual hybridization, producing patterns which simulate DNA microarrays, in order to distinguish between highly-related bacterial strains. In contrast, Squillario et al. [3] investigate interpretability of microarray data gene signatures, enhanced by prior knowledge. The authors contrast a knowledge-driven variable selection (KDVS) tool, with the well-established method of gene enrichment analysis, in order to characterize functionality in the context of Parkinson’s disease data, demonstrating that KDVS provides more effective enhancement. Also concerned with upstream analysis and interpretation of microarray data by enhanced methods, Koschmann and co-authors [4] describe an integrated promoter-pathway analysis approach, which permits causal analysis of co-expressed genes, with potential common regulatory influences. Knowledge-based analysis of the upstream pathway is combined with promoter analysis to obtain hypothetical master regulators, using novel gene expression triclusters, where such regulators link to tumorigenic and apoptotic processes.

The integration of microarray data from multiple datasets, with a view to reduction of noise and enhanced inference is directly addressed by the paper of Sirbu et al. [5] who also note that additional insight from microarrays is gained on features not directly targeted by sequencing methods. The authors present an integration test case, based on public *Drosophila melanogaster* datasets and evaluated using an evolutionary computation framework. They demonstrate that integrative analyses can recover transcriptional gene regulatory networks as well as indicating data types important for both quantitative and qualitative network inference. Also focusing on combined analysis, the work of both Barat et al. [6] and Valavanis et al. [7] considers augmentation of gene expression information through incorporation of DNA methylation data, where abnormal values of the latter are known to be important in cancer onset and development. Barat et al. use publicly available microarray-based gene expression and methylation datasets for colon cancer, to associate locus-specific methylation groups and gene-expression subtypes. Methylation-based subgroups, determined by unsupervised clustering methods, are annotated with expression-based classifications to provide additional information on fine-grained subtype distinctions, important to disease progression. Meanwhile, Valavanis et al. propose an intelligent framework to exploit DNA methylation profiling, in an effort to correlate epidemiological genome scale methylation patterns with cancer (specifically, breast cancer) predisposition. The authors compare evolutionary algorithms and semantic analysis methods in selection of predictive cancer epigenetic biomarkers from large-scale data on methylation measurements at CpG sites, and assess their classification accuracy. Finally, Wang et al. [8] overview algorithms for network component analysis (NCA) as a basis for transcription regulatory network inference, using both combined data from microarrays and ChIP-on-chip as well as prior information about transcription factor gene regulation. Computational principles of NCA, together with limitations and potential solutions are discussed by the authors.

In summary, the papers in this Special Issue provide a selective overview of computational modelling and analysis techniques used to interpret gene expression and related data for a range of biological investigations. Despite the analytical challenges, indications are that microarray measurements, both enhanced and combined with other data types or information, will continue to contribute to the understanding of organism function and disease development for some time to come.
